# Naturally transgenic plants and the need to rethink regulatory triggers in biotechnology

**DOI:** 10.3389/fbioe.2025.1600610

**Published:** 2025-06-30

**Authors:** Danilo Fernández Ríos, Nidia Benítez Candia, Silverio Andrés Quintana, María Florencia Goberna, Eva Nara Pereira, Andrea Alejandra Arrúa, Andrés Castro Alegría

**Affiliations:** ^1^ Facultad de Ciencias Exactas y Naturales, Universidad Nacional de Asunción, San Lorenzo, Paraguay; ^2^ Doctorado en Ciencias Agrarias, Universidad San Carlos, Asunción, Paraguay; ^3^ Coordinación de Innovación y Biotecnología, Dirección Nacional de Bioeconomía, Subsecretaría de Producción Agropecuaria y Forestal, Secretaría de Agricultura, Ganadería y Pesca, Buenos Aires, Argentina; ^4^ Departamento de Biología Molecular y Biotecnología, Instituto de Investigaciones en Ciencias de la Salud, Universidad Nacional de Asunción, San Lorenzo, Paraguay; ^5^ Mycology Investigation and Safety Team, Centro Multidisciplinario de Investigaciones Tecnológicas, Universidad Nacional de Asunción, San Lorenzo, Paraguay

**Keywords:** naturally transgenic plants, horizontal gene transfer (HGT), cellular T-DNA (cT-DNA), *Agrobacterium*, regulatory triggers

## 1 Introduction

Horizontal gene transfer (HGT) is a widespread phenomenon across all domains of life, and has been a driving force of evolution (Keeling and Palmer, 2008; Boto, 2010; Wickell and Li, 2020). Viral sequences have been found in all eukaryotic (Liu et al., 2011; Gilbert and Cordaux, 2013; Takemura, 2020) and prokaryotic kingdoms (Schleper et al., 1992; Rambo et al., 2022), and HGT has been found to occur in all directions between kingdoms of the same domain (Nelson et al., 1999; Keeling, 2009; Fuchsman et al., 2017).

Plant species have stably integrated foreign sequences into their genomes. This natural transgenesis has occurred repeatedly in the evolution of plants, affecting their biology and genetic diversification (Ma et al., 2022). Some of the mechanisms of natural HGT have been characterized to sufficient extent to be used for genetic engineering applications, and the list of mechanisms of gene transfer mastered and applied to engineering might expand with the advancement of scientific knowledge. In order to make the case that natural HGT must be taken into account when designing regulatory frameworks for transgenic organisms, and in particular of transgenic crops, we will address the particular case of HGT from bacteria to plants.

The term “transgenic” usually refers in the literature to DNA constructs resulting from the process of gene transfer between species through genetic engineering (Gordon and Ruddle, 1981; Horsch et al., 1985). *Agrobacterium* ^1^-mediated transformation has established itself as the most widely used method for this purpose. In this procedure, a modified *Agrobacterium* plasmid transfers the desired DNA into the recipient cell, integrating it into its genome and allowing its hereditary transmission (Gelvin, 2009).

## 2 Horizontal gene transfer in plants

For stable incorporation of a sequence into a host organism and its transmission to offspring, certain conditions must be met. First, the foreign sequence must be integrated into the host genome. Then, the incorporated sequence must not be lost in the genomic rearrangements during cell divisions. In addition, the transformed cell must be part of the germline, to ensure inheritance. Finally, the integrated sequence must persist throughout evolution (Lacroix and Citovsky, 2016).

HGT is a process by which genes are transferred between unrelated organisms, as opposed to inheritance from parents. A clear example of gene acquisition by HGT is nitrogen fixation, a metabolic process present in certain bacteria of the genus *Paenibacillus* and regulated by the nif (nitrogen fixation) operon. These metabolic pathways are not specific to *Paenibacillus*, but have been acquired from phylogenetically distant organisms, including some of the Archaea domain and closely related bacterial phyla. HGT plays a key role in these changes, which has resulted in great diversity in the sequence and structure of nitrogen fixation regulatory elements, reflecting the multiplicity of such events from different donor organisms (Fuchsman et al., 2017). Although this phenomenon has been widely documented in Bacteria and Archaea, it has also been observed in eukaryotes, including plants (Keeling, 2024). In the latter, one of the most studied examples of HGT is the transfer of DNA from bacteria of the genus *Agrobacterium* to various plant species (Matveeva, 2021b).

*Agrobacterium* can transfer part of its DNA (T-DNA) to plant cells. Once incorporated, this T-DNA is integrated into the recipient genome, resulting in naturally occurring transgenic plants, or naturally occurring genetically modified plants (nGMs) (Matveeva and Otten, 2019). These plants have sequences in their genomes called cellular T-DNA (cT-DNA), homologous to *Agrobacterium* T-DNAs (White et al., 1983).

Most cT-DNAs identified to date appear to originate from *Agrobacterium rhizogenes*. However, cT-DNAs have also been found with previously unknown T-DNA sequences or unusual combinations thereof (Matveeva and Otten, 2019).

T-DNA sequences naturally transferred by various *Agrobacterium* species contain two types of genes, both regulated by promoters compatible with expression in eukaryotic cells. The first group of genes, called “oncogenes”, encodes proteins that regulate the biosynthesis or response of plant cells to phytohormones, particularly auxins and cytokinins. Their expression causes uncontrolled cell division, leading to tissue proliferation and the formation of neoplastic growths, known as crown galls (De Cleene and De Ley, 1976; Lacroix and Citovsky, 2016). The second group of genes encodes enzymes involved in the synthesis of opines that can be used by *Agrobacterium* cells as a source of carbon and nitrogen (Lacroix and Citovsky, 2016). It has been proposed that for the emergence of a natural transgenic plant, two conditions must be met: the naturally infected plant must be able to regenerate from tissues transformed upon infection; and the structure of the incorporated T-DNA must allow or favor such regeneration (Otten, 2016).

## 3 Evidence of natural transgenesis in plants

HGT in plants was initially identified in species of the genus *Nicotiana*, in whose genomes the presence of *Agrobacterium* T-DNA was detected (White et al., 1983). Studies in *N. glauca* and *N. sylvestris* showed that bacterial DNA insertion was not an isolated event (Khafizova and Matveeva, 2022).

The identification of new cT-DNA sequences in several plant species has been possible thanks to whole genome sequencing databases. The evidence suggests that HGT from bacteria to plants is a more common phenomenon than previously thought and that it has occurred in multiple plant lineages (Bogomaz et al., 2024) (Table 1).

**TABLE 1:** Examples of nGM plants reported in the literature.

Family	Species	References
Apocynaceae	*Apocynum venetum*	Lipatov et al. (2022)
Burseraceae	*Boswellia sacra*	Matveeva (2021a)
Caprifoliaceae	*Lonicera japonica*	Lipatov et al. (2022)
	*Lonicera maackii*	Lipatov et al. (2022)
Caryophyllaceae	*Silene noctiflora*	Matveeva (2021a)
	*Silene uniflora*	Matveeva and Otten (2019), Matveeva (2022)
Convolvulaceae	*Cuscuta australis*	Zhang et al. (2020)
	*Cuscuta campestris*	Zhang et al. (2020)
	*Cuscuta gronovii*	Zhang et al. (2020)
	*Cuscuta suaveolens*	Zhang et al. (2020)
	*Ipomoea batatas*	Kyndt et al. (2015)
	*Ipomoea trifida*	Matveeva and Otten (2021)
Ebenaceae	*Diospyros lotus*	Matveeva (2021a)
Elaeagnaceae	*Elaeagnus angustifolia*	Lipatov et al. (2022)
Ericaceae	*Vaccinium corymbosum*	Matveeva (2021a)
	*Vaccinium macrocarpon*	Matveeva and Otten (2019), Zhidkin et al. (2023)
	*Vaccinium microcarpum*	Matveeva (2023)
	*Vaccinium oxycoccos*	Zhidkin et al. (2023)
Erythroxylaceae	*Erythroxylum cataractarum*	Zhidkin et al. (2023)
	*Erythroxylum daphnites*	Matveeva (2022)
	*Erythroxylum densum*	Lipatov et al. (2022)
	*Erythroxylum havanense*	Matveeva (2022)
Euphorbiaceae	*Triadica sebifera*	Matveeva (2022)
Fabaceae	*Aeschynomene evenia*	Matveeva (2021a)
	*Arachis appressipila*	Yugay et al. (2025)
	*Arachis macedoi*	Bogomaz et al. (2024)
	*Arachis magna*	Bogomaz et al. (2024)
	*Arachis monticola*	Yugay et al. (2025)
	*Arachis paraguariensis*	Bogomaz et al. (2024)
	*Arachis pintoi*	Yugay et al. (2025)
	*Arachis pusilla*	Bogomaz et al. (2024)
	*Arachis rigonii*	Bogomaz et al. (2024)
	*Arachis stenophylla*	Yugay et al. (2025)
	*Arachis stenosperma*	Bogomaz et al. (2024)
	*Arachis trinitensis*	Yugay et al. (2025)
	*Arachis valida*	Bogomaz et al. (2024)
	*Arachis villosa*	Yugay et al. (2025)
	*Eperua falcata*	Matveeva (2021a)
Kewaceae	*Kewa caespitosa*	Matveeva (2021a)
Myrtaceae	*Eucalyptus cloeziana*	Matveeva (2021a)
Molluginaceae	*Pharnaceum exiguum*	Matveeva (2021a)
Nyssaceae	*Nyssa sinensis*	Matveeva (2021a)
Paulowniaceae	*Paulownia fortunei*	Lipatov et al. (2022)
Plantaginaceae	*Linaria acutiloba*	Matveeva and Lutova (2014)
	*Linaria dalmatica*	Vladimirov et al. (2019)
	*Linaria genistifolia* subsp. *dalmatica*	Matveeva and Lutova (2014)
	*Linaria vulgaris*	Matveeva and Lutova (2014)
Rhizophoraceae	*Ceriops decandra*	Matveeva (2022)
Salicaceae	*Populus alba × Populus glandulosa*	Matveeva (2021a)
Solanaceae	*Nicotiana glauca*	Suzuki et al. (2002)
	*Nicotiana noctiflora*	Zhidkin et al. (2023)
	*Nicotiana otophora*	Matveeva and Lutova (2014)
	*Nicotiana sylvestris*	Matveeva and Lutova (2014), Khafizova and Matveeva (2022)
	*Nicotiana tabacum*	Suzuki et al. (2002)
	*Nicotiana tomentosa*	Matveeva and Lutova (2014)
	*Nicotiana tomentosiformis*	Matveeva and Lutova (2014)
Theaceae	*Camellia oleifera*	Lipatov et al. (2022)

Genes acquired by HGT can retain their functionality in recipient plants and influence their traits. An example of this is *Ipomoea batatas*, where a cT-DNA has been identified with functional *Agrobacterium* genes which have remained stable over time (Kyndt et al., 2015). In addition, such genes can affect certain phenotypic traits, such as the *rol* genes, associated with root development (Quispe-Huamanquispe et al., 2017).

Unlike transgenics obtained through genetic engineering, in which genes are inserted in a targeted manner in the laboratory, nGMs acquired foreign DNA through natural infections (Chen and Otten, 2017). Between 5%–10% of dicotyledonous species are estimated to contain cT-DNAs (Matveeva, 2021b). With approximately 200 million species in this class, about 10,000 species would be nGM plants (Folta and Otten, 2021). The existence of nGM plants challenges the separation between “natural” and “artificial” made by regulatory triggers when determining which types of plants should be subjected to biosafety assessments, by showing that transgenics are not only the result of human manipulation, but also a naturally occurring phenomenon.

## 4 Discussion

The natural presence of *Agrobacterium* sequences in plant organisms questions the logic of strictly regulating transgenics obtained through genetic engineering, while exempting organisms that are similar, but obtained through conventional methods (Ammann, 2014; McHughen, 2016). cT-DNA evidence suggests that regulation focused on the method of production may be inadequate (Gould et al., 2022). In many regulatory frameworks, a transgenic organism is one that contains deliberately altered genetic material which does not occur “naturally” through breeding or selection (EFSA, 2024). This inconsistency becomes more evident when considering that the same trait can be obtained both by genetic engineering techniques and by conventional breeding, creating different regulatory thresholds for products with the same traits.

These inconsistencies also extend to relevant aspects of risk assessment, given that HGT represents an important topic in the evaluation of GM plants. In regulatory practice, HGT is typically evaluated using a pathway-to-harm approach (OECD, 2023). However, to date, no empirical evidence supports HGT from GM plants to soil bacteria under field conditions (Badosa et al., 2004; Demanèche et al., 2011; Ma et al., 2011). Similarly, while humans and animals routinely ingest DNA from multiple biological sources, the likelihood of HGT from GM plant-derived DNA to gut microbiota or host tissues remains extremely low (Jennings et al., 2003; Netherwood et al., 2004; Sieradzki et al., 2013; Korwin-Kossakowska et al., 2016). A detailed assessment of the potential for HGT from GM plants to microorganisms is beyond the scope of this work. For further information, readers are encouraged to consult Philips et al. (2022) for a detailed review.

Given these complexities, the existence of nGM plants highlights the need for a product-based regulatory trigger in which biosafety assessment focuses on the traits and phenotype of the final organism rather than the process by which it was obtained (McHughen, 2016). This approach offers several important advantages over traditional process-based frameworks, particularly in the context of emerging breeding techniques. Focusing on the characteristics and potential risks of the final product ensures regulatory coherence and risk assessment proportionality, avoiding inconsistencies where crops with similar traits are subject to different oversight (Caccamo, 2023; Brookes and Smyth, 2024). Not all GMOs pose the same level of risk; some have well-characterized, low-risk profiles; just as not all conventionally bred crops are inherently safe. Conventional methods such as wide crosses, mutagenesis, or spontaneous mutations can also result in traits with biosafety implications, including increased toxicity, allergenicity, or invasiveness (McHughen, 2016). While these products are generally not subject to a complete risk assessment, they are often regulated at various stages of the production chain (registration for the crop, safety assessment for the byproducts).

A product-based approach enables regulators to focus their efforts on the actual risk presented by a crop rather than presuming risk based on the technique employed (Sprink et al., 2016). This logic has already been adopted in the case of NBTs by countries such as Argentina, Brazil and Canada, that exclude certain products developed through NBTs from GMO regulations when no novel combination of genetic material is present in the final product (Goberna et al., 2023; Fernandes et al., 2024; Lubieniechi et al., 2025). This aligns with risk assessment principles that prioritize the traits of the crop. It also allows for the inclusion of conventionally bred crops in biosafety assessments when they present novel or potentially hazardous traits, which process-based systems tend to overlook (Gould et al., 2022). Altogether, adopting a product-based perspective would contribute to building a coherent, adaptable, and science-driven regulatory framework for novel organisms.

## Footnotes

1 The collective term *Agrobacterium* is used in recognition of tradition and due to the impossibility of precisely identifying the bacterium responsible for the plant transformation that occurred millions of years ago. The T-DNA fragments present in plant genomes are insufficient for this determination (Matveeva, 2021a). The classification of the genus is still evolving, as the taxonomic affiliation of five *Agrobacterium* genomospecies has not yet been determined, suggesting a possible increase in the number of species in the future (Flores-Félix et al., 2020).
